# Magnitude of maternal near-misses and associated factors in Arsi Zone public hospitals in Oromia, Ethiopia, 2022

**DOI:** 10.1016/j.heliyon.2024.e24910

**Published:** 2024-01-18

**Authors:** Wogene Morka Regassa, Getu Megersa Gemeda, Elias Bekele Wakwoya, Bedasa Woldemichaele Gelete

**Affiliations:** a Department of Midwifery, College of Health Sciences, Arsi University, Asella, Ethiopia; b Department of Nursing, College of Health Sciences, Arsi University, Asella, Ethiopia

**Keywords:** Maternal near-miss, Public hospital, Women, Morbidity, Mortality, Arsi Zone, Oromia, Ethiopia

## Abstract

**Background:**

Investigation of maternal near-misses is useful for monitoring and evaluating the quality of obstetrics care services. Despite its importance, data has been limited in Arsi Zone public hospitals.

**Objective:**

To assess the magnitude of maternal near-miss and associated factors in Arsi Zone public hospitals, Ethiopia, 2022.

**Method:**

Institution-based cross-sectional study design was conducted on 327 study participants from December 2021 to June 2022. The study participants were selected through systematic random sampling. Trained data collectors used pre-tested structured questionnaires to collect data from study participants. Pertinent data were also extracted from clients’ logbook. The data were entered to Epi Data version 3.1 and exported to SPSS version 25.0 for analysis. Multivariable logistic regression were employed to control for possible confounders where a significance level was set to a P-value of 5 %.

**Result:**

A total of 326 study participants responded, resulting in a 99.7 % response rate. The magnitude of maternal near-miss was 34.4 % [95 % CI (29.2–39.8)]. Hypertensive disorders (35 %), hemorrhage (35 %), ruptured uterus (11 %), unsafe abortion (8 %), obstructed labour (7 %), and infection/sepsis (4.5 %) were the direct while anemia (20 %) was one of the indirect causes of maternal near-misses. ANC visit received (AOR = 2.5, 95 % CI: 1.04–5.84), First ANC booked trimester (AOR = 0.26, 95 % CI: 0.1–0.9), delay in seeking care (AOR = 3.1, 95 % CI: 1.2–8.1), delay two (AOR = 2.7, 95 % CI: 1.0–6.8) and mode of delivery (AOR = 2.8, 95 % CI: 1.3–6.1) were factors associated with maternal near-misses.

**Conclusion:**

The prevalence of maternal near-miss was high. To improve the identified factors and minimize their consequences, appropriate interventions are required at all levels to improve the quality of obstetrics care services aimed at improving positive pregnancy outcomes.

## 1 Introduction

Maternal mortality is a global health concern. Every day, 800 women die in the world from the preventable complications related to pregnancy, childbirth, and post-natal periods [1]. It has also been documented that for every woman who dies, approximately twenty others suffer from severe morbidity [2]. Despite showing promise in decreasing, maternal mortality remains a top priority for the world, and the Sustainable Development Goal (SDG) aims to reduce it to less than 70 per 100,000 live births by 2030 [3].

Sub-Saharan Africa has the highest mortality rate, with 533 deaths per 100,000 live births, or 200,000 deaths per year. Southern Asia comes in second, with 163 maternal deaths per 100,000 live births, or 57,000 maternal deaths per year. Ethiopia is one of 15 countries with a 'high alert' maternal death rate of 401 per 100,000 live births [1,4,5]. Although maternal mortality trends in Ethiopia have been decreasing, from 871 in 2006 to 412 per 100,000 live births in 2016, it remains the highest in the world [4].

Contrary to this, nowadays, maternal death in a health facility is rare, leading to an insignificant number. To address this gap, maternal near-miss has been proposed as a supplement to maternal death. It is defined as "a woman who nearly died but survived a complication during pregnancy, childbirth or 42 days after termination of pregnancy". The World Health Organization (WHO) developed the concept assists health systems in evaluating and improving the quality of obstetric care. Despite having similar pathological and circumstantial factors, some women die, and others narrowly escape death [6]. Fortunately, a woman who survives the complications serves as a surrogate, enabling us to gain a better understanding of the preventable factors that contribute to maternal death [7]. A woman may survive complications either due to chance or the quality of care provided. Hence, investigating maternal near-misses provides a more comprehensive assessment of the quality of obstetric care services [8].

Previous studies reported that the magnitude of maternal near-misses varies across countries; the highest rate is in low and middle income countries [[9], [10], [11], [12], [13]]. It ranges from 2.2 per 1000 live births in Malaysia to 94.1 per 1000 live births in South Sudan [11,14]. In Ethiopia, it also ranges from 4.97 to 92.1 per 1000 live births [15,16]. Despite the presence of a few studies on maternal near-misses [12,15,17,18],critical variables such as maternal nutritional status and delays were missing, in addition to a paucity of data in the study setting. Thus, the study provides important evidence related to obstetrics care services, which may help reduce maternal near-misses. It also helps to fill the knowledge gap and provide reliable evidence for policymakers, programmers, and health practitioners. Therefore, this study aimed to assess the magnitude and factors associated with maternal near-misses in Arsi Zone public hospitals.

## 2 Materials and methods

### 2.1 Study design, setting and period

From December 2021 to June 2022, an institution-based cross-sectional study design was employed at public hospitals in Arsi Zone, Oromia regional state, Ethiopia. The Zone has 498 health posts, 240 private clinics, 107 government health centers, and 8 government hospitals, namely; Asella Teaching and Referral Hospital, Kersa Primary Hospital, Abomsa Primary Hospital, Gobessa Primary Hospital, Arsi Robe Primary Hospital, Bekoji Primary Hospital, Sire Primary Hospital, and Belegesgar Primary Hospital. Except for Bekoji Primary Hospital, which has been closed for COVID-19 treatment during the data collection period, the rest provide maternal health and newborn care services.

### 2.2 Source population and study participants

All women who were admitted to hospitals during pregnancy, labour and delivery and/or within 42 days of delivery or termination of pregnancy were the source population. All sampled women who were managed at selected hospitals during pregnancy, labour and delivery and/or within 42 days of delivery or termination of pregnancy were study participants.

### 2.3 Operational definition

All women who met at least one of the WHO disease-validated criteria listed below were eligible for a maternal near-miss during pregnancy, labor and delivery, and/or within 42 days of delivery or termination of pregnancy. A woman was said to have a maternal near-miss event if she met one of the WHO disease-validated criteria, such as: obstructed labour(uterine rupture, impeding rupture like prolonged labour with previous C/S, emergency C/S), hemorrhage (severe obstetrics hemorrhage leading to shock, emergency hysterectomy, coagulation defects, and/or blood transfusion of at least one unit), pregnancy induced hypertension disorders(severe pre-eclampsia, eclampsia), sepsis (septic abortion, infections including hypothermia or hyperthermia or a clear source of infection and clinical signs of septic shock), and severe anemia (including low hemoglobin <6 g/dL or clinical signs of severe anemia in woman without hemorrhage) [6]. In accordance with the aforementioned criteria, resident doctors in the wards identified near-miss events. A woman with accidental or incidental causes and revisiting during the study period were all excluded.

### 2.4 Sample size and sampling technique

**Fig. 1:**
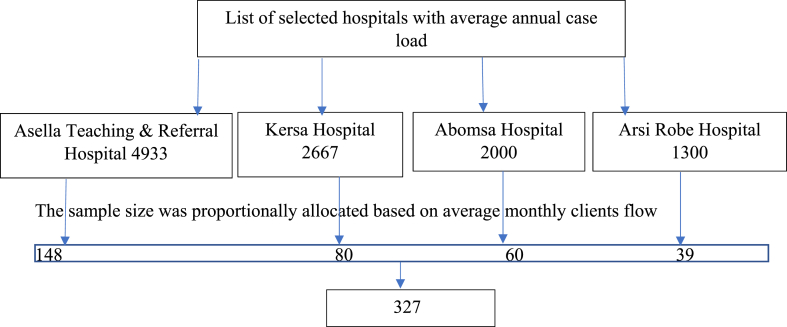
Schematic representation of sample size allocation to participated hospitals in Arsi Zone, Ethiopia, 2022.

The sample size was determined using the single population proportion formula by taking the previous prevalence of maternal near-miss 73.8 [8], P-value = 0.05, margin of error = 0.05, and 10 % non-response rate. The total sample size was 327. Based on the number of cases, the desired number of study participants has been allocated at each hospital using a proportionate sampling method. The preceding averaged 908 women monthly divided by sample size, resulting in a K^th^ value of approximately 3. Systematic sampling method was implemented at each hospital to select every third of the study participants from a log book. Due to logistical, COVID -19 pandemic, and feasibility concerns, we selected four of seven public hospitals at random (Fig. 1).

### 2.5 Data collection tools and collection procedure

Following a thorough review, standard questionnaires were adopted with modifications from previous studies [6,15,[18], [19], [20]]. The contextualized questionnaires were used to collect data on the respondents’ demographics, access to reproductive health services, history of pregnancies, current and past medical conditions, and nutritional status. Additionally, information was abstracted from medical records using data extraction tools modified from WHO standard tools. Four independent data collectors who were not working at the selected hospital and one supervisor assigned. After completing and signing each questionnaire, the data collectors had their work checked by supervisors.

### 2.6 Data quality assurance

The questionnaires were designed and modified in English and then translated to the local language, Afan Oromo. To ensure the consistency of the questionnaires, they were translated back to English by another language expert. The data collection tool was pre-tested on 5 % of the sample size at Adama Hospital-located outside of the study area. Some of the items were modified based on the results of the pre-test. Data collectors and supervisors received a day-long training on how to use the tool, maintain ethical principles and screen maternal near-miss conditions using WHO disease-validated criteria. The supervisors monitored the data collection process to assure the quality of the data. Daily, the field supervisor checked the completeness of the collected data. Before data entry, the completeness, accuracy, and consistency of the data were checked. Incomplete questionnaires were excluded from the analysis. The interview was conducted privately. Furthermore, data was entered into Epi Data, exported to SPSS and checked for outliers.

### 2.7 Data management and analysis

Epi Data version 3.1 was used for data entry and cleaning, and SPSS version 25 was used for analysis. Descriptive analysis was done to determine the magnitude of maternal near-misses using denominators per 1000 live births. The maternal mortality index was analyzed and expressed as a percentage. Bivariate analysis was used to examine the relationship between the dependent and independent variables, and an odds ratio with a 95 % confidence interval was analyzed. All variables with a P-value of 0.25 in the bivariate analysis were included in the multivariate logistic regression analysis model to depict factors related to dependent variables. The P-value for statistical significance was set at 5 %. The multicollinearity test was assessed to check the correlation between independent variables using variance inflation factor (VIF) and no multicollinearity was detected. The VIF score was less than 4. The model fitness was determined using the Hosmer-Lemeshow goodness-of-fit test (P = 0.61).

### 2.8 Ethical consideration

After the study proposal was approved by the review board committee of Arsi University's College of Health Sciences, ethical approval was obtained under protocol number A/CHS/RC/60/2022. Permission letters were obtained from the Arsi Zone Health Department Office. A letter of support was sent to all concerned bodies. After explaining the study's purpose, duration, potential risks, and benefits, study participants provided informed verbal consent.

## 3 Result

### 3.1 Magnitude of maternal near-misses

**Fig. 2:**
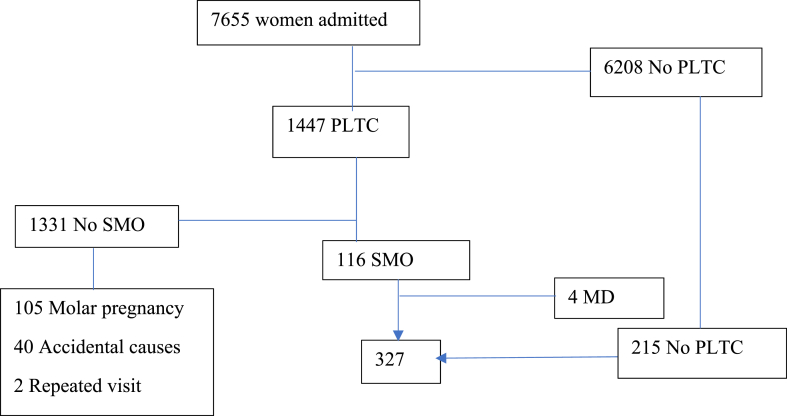
Flow diagram showing identification processes of required sample sizes.

A total of 7655 women were admitted to four hospitals during the study period; of these, 7475 live births, 1447 potential life-threatening complications (PLTC), 31 stillbirths, and 4 maternal deaths (MD) took place. Out of 327 women, 326 (99.7 %) agreed to participate in the study. One hundred twelve women were on the verge of death but survived. Hence, the prevalence of maternal near-miss was 112 (34.4 %) [(95 % CI 29.2–39.8)] whereas the maternal mortality index was 4 (3.4 %) (Fig. 2).

**Fig. 3:**
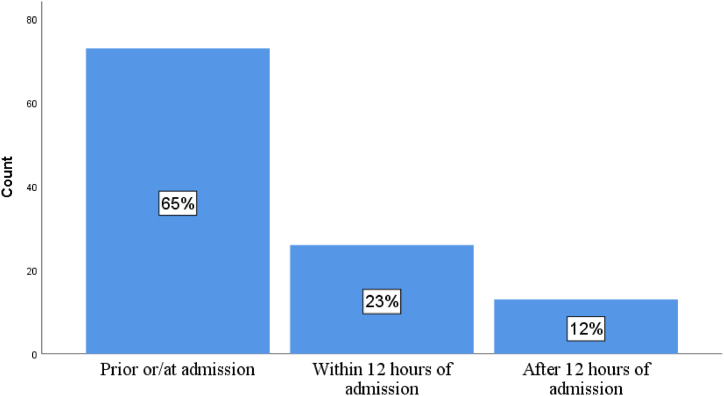
Time of maternal near-miss occurrence in Arsi Zone public hospitals, Ethiopia, 2022.

**Rate of time of maternal near-miss occurrence** (Fig. 3).

### 3.2 Socio demographic characteristics of the study participants

The age of participants range from 17 to 42 years, with the mean age 26.3 and standard deviation (SD) ±4.9 years (Table 1).

**Table 1:** Socio-demographic characteristics of study participants in Arsi Zone, public hospitals, Ethiopia, 2022.

Variables	Frequency	Percent
Age in years		
15–24	114	35
25–29	124	38
30+	88	27
Religion		
Christian	150	46
Muslim	176	54
Marital status		
Married	315	97
Unmarried	11	3
Early marriage status		
Yes	47	14
No	279	86
Educational status		
Cannot read & write	43	13
Primary	117	36
Secondary	120	37
Tertiary	46	14
Women occupation		
Employed	233	72
Unemployed	93	28
Partners occupation (n = 315)		
Government employee	89	28
Private employee	120	38
Farmer	106	34
Monthly average income (mean = 4864.5, SD = ±3413.8)		
<2000	86	26
2001–6000	150	46
>6001	90	28
Place of residence		
Urban	165	51
Rural	161	49

### 3.3 Obstetrics and reproductive characteristics of study participants

The greater percentage of study participants—304 (93 %)—received antenatal services during their current pregnancy. The majority 127 (56 %) of the women who gave birth had a pregnancy interval of less than two years. Greater proportions of the women experienced spontaneous vaginal delivery 173(53 %) (Table 2).

**Table 2:** Obstetrics and Reproductive characteristics of study participants in Arsi Zone public hospitals, Ethiopia 2022.

Variable		Frequency	Percent
**Received ANC**	Yes	304	93
	No	22	7
**Number of ANC received (N = 304)**	One	7	2
	Two	61	20
	Three	90	30
	Four & above	146	48
**First ANC visit booked at (N = 304)**	First trimester	115	38
	Second trimester	133	44
	Third trimester	56	18
**History of pregnancy**	Yes	229	70
	No	97	30
**History of abortion(N = 229)**	Yes	85	37
	No	144	63
**Gravidity (N = 229)**	Primipara	28	12
	Multipara	148	65
	Grandpara	53	23
**Number of alive child(N = 229)**	No child (0)	9	3
	1–2	209	64
	3–4	67	20
	>4	41	13
**Pregnancy interval (N = 229)**	<24 months	127	56
	≥24 months	102	44
**Mode of delivery**	SVD	173	53
	AVD	26	8
	C/S	89	27
	Instrumental	31	10
	Other	7	2

**Maternal nutritional status and reproductive health services** (Table 3).

**Table 3:** Distribution of nutritional status and accessibility to reproductive health services of study participants in Arsi Zone public hospitals, Ethiopia 2022.

Variables		Frequency	Percent
**Nutritional status (BMI)**	Underweight	14	4
	Overweight	74	23
	Normal	238	73
**No delay in making decision**	Yes	249	76
	No	77	24
**No delay to go to hospital**	Yes	243	75
	No	83	25
**Reason for delay to go to hospital (N = 83)**	Lack of transport	46	55
	Lack of money	15	18
	Bad road condition	14	17
	No facility nearby	5	6
	Other	3	4
**Travel time to arrive at hospital**	<1 h	194	60
	>1 h	132	40
**Waiting time before treatment**	<30	308	95
	>30	18	5
**Problem faced at hospital**	Delay in making correct diagnosis	4	1
	Delay in providing treatment	28	9
	Lack of supply and equipment	42	13
	Poor monitoring of patient	32	10
	No problem faced	220	68
**Admission mode**	Self	145	44
	Referred	181	56
**Referred from (N = 181)**	Hospital	12	7
	Health center	134	74
	Private health facility	35	19
**Mode of transport**	Ambulance	153	47
	Others^a^	173	53

a Public transport, personal vehicles, Bajaj, Carts (Gaarii).

### 3.4 Causes of maternal near-misses

**Indirect causes of maternal near-misses** (Table 4).

**Table 4:** Indirect causes of maternal near-miss in Arsi Zone public hospitals, Ethiopia, 2022.

Variables	Frequency	Percent
**Anemia**	23	20.5
**Cardiac problems**	3	2.7
**No indirect causes**	86	76.8
**Total**	112	100

**Fig. 4:**
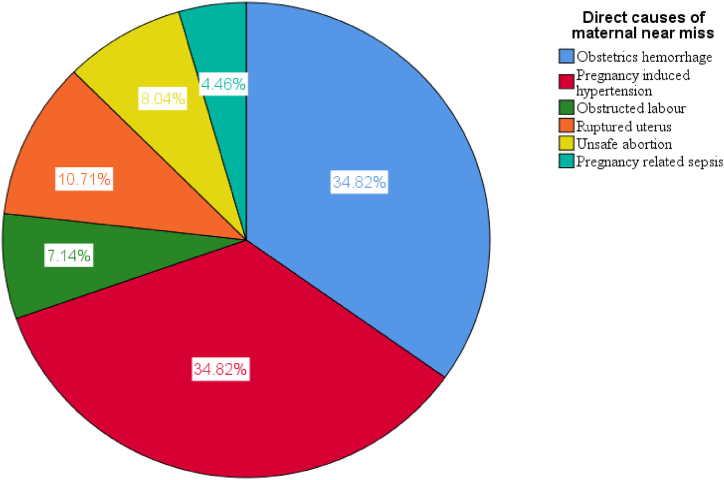
Distribution of direct causes of maternal near-miss in Arsi Zone public hospitals, Ethiopia, 2022.

**Distribution of direct causes of maternal near-misses** (Fig. 4).

### 3.5 Factors associated with maternal near-misses

In binary logistic regression analysis, variables significantly associated with maternal near-miss were transferred to multivariate logistic regression analysis to control for confounding factors. After controlling for confounding variables, the number of ANC contacts received, the first ANC booked in the third trimester, the mode of delivery, the delay in seeking care, and the delay two were found to be substantially linked with maternal near-misses (Table 5).

**Table 5:** Logistic regression analysis of selected variables and maternal near-miss in Arsi Zone public hospitals, Ethiopia, 2022.

Variables with category		Maternal near-miss		COR (95 % CI)	AOR (95 % CI)	P-Value
		Yes (%)	No (%)			
Educational status	Can't read & write	11 (10)	32 (15)	0.5(0.2–1.5)	3.3(0.6–17.6)	0.17
	Primary (1–8)	58 (52)	59 (28)	**0.2(0.1–0.4)**	0.5(0.1–2)	0.37
	Secondary (9-12)	36 (32)	84 (39)	0.4(0.2–1.0)	1.2(0.3–4.6)	0.80
	Tertiary	7 (6)	39 (18)	1	1	
Residence	Rural	39 (35)	122 (57)	**2.5(1.5–3.9)**	0.8(0.3–2)	0.56
	Urban	73 (65)	92 (43)	1	1	
Admission mode (Referral)	Self (No)	34 (30)	111 (52)	**2.5(1.5–4)**	1(0.4–2.7)	0.98
	Referred (Yes)	78 (70)	103 (48)	1	1	
Women's occupation	Employed	88 (79)	145 (72)	1	1	
	Unemployed	24 (21)	69 (32)	**1.7(1.0–2.9)**	1.1(0.4–3)	0.83
Average monthly income	<2000	41 (37)	45 (21)	1	1	
	2001–6000	48 (43)	102 (48)	**1.9(1.1–3.3)**	1.4(0.6–3.4)	0.42
	>6001	23 (20)	67 (31)	**2.7(1.4–5.0)**	1.6(0.5–5)	0.44
Number of ANC received (N = 304)	≥4	76 (76)	82 (40)	1	1	
	≤3	24 (24)	122 (60)	**4.7(2.75–8.1)**	**2.5(1.04–5.84)**	**0.041**
ANC visit booked at (N = 304)	1st trimester	24 (24)	91 (45)	1	1	
	2nd trimester	53 (53)	80 (39)	**0.4(0.2–0.7)**	0.7(0.3–1.7)	0.39
	3rd trimester	23 (23)	33 (16)	**0.4(0.2–0.8)**	**0.26(0.1–0.9)**	**0.032**
Number of alive child	No child	24 (21)	73(34)	1	1	
	One child	45(40)	76(36)	0.56(0.3–1)		
	Two children	16(14)	51(23)	1.0(0.5–2.2)	1.5(0.6–4)	0.32
	Three children	27(24)	14(7)	**0.17(0.1–0.4)**	0.6(0.2–1.8)	0.36
Pregnancy interval (N = 229)	<24 months	58 (66)	69 (49)	**0.5(0.3–0.9)**	0.5(0.2–1.1)	0.08
	≥24 months	30 (34)	72 (51)	1	1	
Mode of delivery	SVD	40 (38)	133(62)	**2.7(1.6–4.3)**	**2.8(1.3–6.1)**	**0.012**
	Others^b^	65(62)	81(38)	1	1	
Delay in seeking care	Yes	70 (63)	179 (84)	**3(1.8–5.2)**	**3.1(1.2–8.1)**	**0.018**
	No	42 (37)	35 (16)	1	1	
Once you decided did you go straight.	Yes	63(56)	180 (84)	1	1	
	No	49(44)	34 (16)	**0.24(0.14–0.4)**	1.3(0.5–3.2)	0.57
Time to reach a hospital	<1 h	46(41)	148(69)	1	1	
	>1 h	66(59)	66(31)	**3.2(2.0–5.2)**	**2.7(1.0–6.8)**	**0.043**
Mode of transport	Ambulance	71(63)	82(38)	1	1	
	Others^a^	41(37)	132(62)	**2.8(1.7–4.5)**	1.5(0.6–3.7)	0.39

a Public transport, personal vehicles, Bajaj, cart. Bold indicates: P-value <0.05.

b C/S, Assisted vaginal delivery, complete abortion, and instrumental delivery.

## 4 Discussion

The study sought to determine the magnitude and factors associated with maternal near-misses in Arsi Zone public hospitals. Hence, the prevalence of maternal near-miss was 34.4 % (95 % confidence interval [CI] 29.2–39.8).The finding is comparable to the study conducted in Ghana [12], but greater than other African countries and India [9,11,21,22], and less than South Sudanese [14].These variations could be attributed to underutilization of maternal health services, delay in seeking care due to low literacy, low awareness, poor recognition of danger signs by the women, family members, health centers, and inadequate transportation facility [23]. In this study, approximately two-thirds 73 (65 %) of the women with maternal near-miss cases were admitted already in a near-miss status, implying a delay in seeking care.

Unlike earlier studies conducted in some parts of Ethiopia, such as West Ethiopia (4.97 %), Bale Zone (28.7 %), Amhara Regional Referral Hospitals (23.3 %), University of Gondar Hospital (15.8 %), and Debre Markos Referral Hospital (29.7 %) [13,17,[24], [25], [26]], this finding is high. This might be because of low health seeking behavior of study participants and study settings. Another possible explanation might be nearly half of the study participants −161 (49 %)—were from rural areas. It could be linked to a delay in receiving early treatment for complications, resulting in maternal near-misses. It can be argued that well-equipped facilities, skilled personnel, and an efficient working system can contribute to averting severe disability and death [14].

On the other hand, the current prevalence is lower than the prevalence reported in studies conducted at Jimma University Referral Hospital (59.2 %) and in the Eastern part of Ethiopia 92.1 % [16,27]. The difference is mainly due to study approaches, instruments used, and the type of facility.

The present study showed that the maternal mortality index was 4 (3.4 %). According to WHO recommendation, a maternal mortality index greater than 5 % indicates poor quality of obstetrics complications care, and high maternal mortality exists within hospitals [28]. In this regard, although the current study showed that there is a good quality of obstetrics care, there are different causes and factors associated with maternal near-misses identified in Arsi Zone public hospitals.

Hypertensive disorders of pregnancy 39 (35 %) and severe hemorrhage 39 (35 %) were equally the leading causes of maternal near-miss. Previous studies conducted in middle and high income countries reported that the primary cause of maternal near-miss was hypertensive disorders, followed by severe obstetric hemorrhage [13,16,24,[29], [30], [31], [32]]. However, the current finding is similar to a study conducted in Western Ethiopia and Jimma University Referral Hospital, in that hypertension and hemorrhage were equally the leading causes of maternal near-miss [8,15]. The possible reason for these problems might be indicative of some form of delay in managing obstetric complications at health facilities.

Obstructed labour was also another cause of maternal near-miss which was also revealed in most of the previously done studies in developed countries [24,29,32]. This finding is also in line with other studies conducted in Egypt and different parts of Ethiopia that reported that obstructed labour could cause maternal near-miss [9,17]. This could be explained as the management of obstructed labour should require prompt diagnosis and proper intervention to reduce maternal morbidity. The link between obstructed labour, delay one, and delay two may also be an alternative explanation. Hence, the odds of maternal near-misses increase in the presence of obstructed labour.

In our study, sepsis was one of the least common causes of maternal near-misses. The lower percentage of pregnancy-related infections that cause maternal near-misses may be due to early antibiotic treatment at health facilities.

Anemia was the most common indirect cause of maternal near-misses in our study. The finding is consistent with studies conducted in African countries such as Egypt and Nigeria [7,9] as well as Oromia, Ethiopia [17]. Nutrition and iron deficits, as well as previous malaria, are the main causes of anemia during pregnancy. An investigation into how anemia could be the cause of maternal near-misses is required.

Multivariate logistic regression revealed a statistically significant association between maternal near-miss and ANC visits booked during the third trimester, frequency of ANC visits less than four times, delay in deciding to go to hospital, delay in arriving at hospital on time, and mode of delivery.

The study suggested that a woman who booked ANC during the third trimester of pregnancy was 0.26 times less likely to experience a maternal near-miss. The rate of maternal near-misses decreased by a 74 % among women who started ANC in the third trimester of pregnancy (AOR = 0.26, 95 % CI (0.1–0.9), P = 0.03). The amount of ANC contacts received, as contrasted to the time of ANC initiation, had an impact on a pregnant woman's wellbeing. Thus, a woman who had a history of less than four ANC visits was 2.5 times more likely to have a maternal near-miss than a woman who had more than four ANC visits (AOR = 2.5, 95 % CI (1.04–5.84, P = 0.041). This finding is comparable with the study done in Indonesia, and according to the WHO recommendation [33,34], more ANC visits should benefit the mothers and minimize all delays. Hence, health care providers should advise women to have ANC visits more than the minimum standard, four times.

The study also discovered that delayed decisions to go to a hospital had statistical significance among women who had experienced maternal near-misses. So, a woman who experienced a delay one was three times more likely to experience a maternal near-miss (AOR = 3.1, 95 % CI (1.2–8.1, P = 0.018). Delay in arriving at a hospital was a strong predictor of maternal near-miss. A woman who traveled more than an hour was nearly three times more likely developed maternal near-miss than a woman who traveled less than an hour (AOR = 2.7, 95 % CI (1.0–6.8, P = 0.043). The finding is consistent with previous researches [8,[13], [14], [15],[35], [36], [37], [38]], and complemented by the fact that 73 (65 %) of women experienced maternal near-misses before or immediately after arriving at a hospital. The delay was due to a lack of transportation (55 %), money (18 %), and poor road conditions (17 %). The other reasons may include poor recognition and underestimation of clinical conditions by the women and family members.

A woman who gave birth by spontaneous vaginal delivery was nearly three times more likely developed maternal near-miss (AOR = 2.8, 95 % CI (1.3–6.1, P = 0.012). The finding is comparable with research conducted in Indonesia [33]. A woman who gave birth via SVD was more susceptible to maternal near-miss events. This might be due to poor monitoring of women during labor and delivery, as well as the fact that the phrase ’normal labor’ might be deceptive. This implies that health professionals should strive to minimize negligence originating from the wrong perception about 'normal labour'. We recommend that no labour be considered 'normal,' because no one knows when complications begin to develop during labour and delivery.

## 5 Limitation of the study

The study did not look into maternal near-misses at lower levels of care, such as health centers. Although the majority of women who experienced obstetric complications were frequently referred to hospitals, a small number of women may occasionally seek care from lower-level facilities. As a result, the findings of this study can only be applied to hospitals throughout the country. The mortality index reported in this study was only the numbers recorded in the hospital's logbook. Therefore, the number of deaths may be influenced by maternal deaths at home.

## 6 Conclusion

The study discovered that the magnitude of maternal near-misses in Arsi Zone public hospitals was high. The factors that led to maternal near-misses were: first ANC booked trimester, number of ANC visits received, delay in seeking care, delay two, and mode of delivery. To overcome the identified factors and minimize their consequences, appropriate interventions at all levels need to be strictly implemented to improve the quality of obstetrics care services and positive pregnancy outcomes.

## Funding statement

Not available.

## Data availability statement

The dataset used during this study will be made available on request to the corresponding author.

## CRediT authorship contribution statement

**Wogene Morka Regassa:** Writing – review & editing, Writing – original draft, Visualization, Validation, Supervision, Software, Resources, Project administration, Methodology, Investigation, Formal analysis, Data curation. **Getu Megersa Gemeda:** Writing – review & editing, Validation, Supervision, Software, Methodology, Formal analysis, Data curation. **Elias Bekele Wakwoya:** Writing – review & editing, Validation, Supervision, Software, Methodology, Formal analysis, Data curation. **Bedasa Woldemichaele Gelete:** Writing – review & editing, Visualization, Supervision, Software, Methodology, Formal analysis, Data curation.

## Declaration of competing interest

The authors declare that they have no known competing financial interests or personal relationships that could have appeared to influence the work reported in this paper.

## Abbreviations

ANC: Antenatal care
AOR: Adjusted odds ration
CI: Confidence interval
COR: Crude odd ratio
C/S: Cesarean section
MNM: Maternal near miss
PLTC: Potentially life threatening complication
SVD: Spontaneous vaginal delivery
SMO: Severe maternal outcome
VIF: Variance inflation factor
WHO: World Health Organization
